# Blaťácké Zlato Cheese: A Screenshot of Its Biofunctional and Physicochemical Characteristics

**DOI:** 10.3390/foods14132208

**Published:** 2025-06-23

**Authors:** Sandra T. Martín-del-Campo, Alexa Pérez-Alva, Sheba Sunny-Marottickal, Michaela Freyová, Tomáš Kudera, Iveta Klojdova, Diana K. Baigts-Allende

**Affiliations:** 1 DRIFT-FOOD Research Centre, Faculty of Agrobiology, Food and Natural Resources, Czech University of Life Sciences Prague, Kamýcká 129, 16500 Prague, Czech Republic; perez_alva@af.czu.cz (A.P.-A.); xmarottickal@uniag.sk (S.S.-M.); freyova@af.czu.cz (M.F.); kuderat@af.czu.cz (T.K.); klojdova@af.czu.cz (I.K.); baigts_allende@af.czu.cz (D.K.B.-A.); 2 Danube AgriFood Master Program (DAFM), Faculty of Agrobiology, Food and Natural Resources, Czech University of Life Sciences Prague, Kamýcká 129, 16500 Prague, Czech Republic

**Keywords:** Blaťácké zlato cheese, ABTS, DPPH, total polyphenol content, cheese characterization, color

## Abstract

This study aims to determine the Blaťácké zlato cheese in vitro antioxidant activity and its correlation with specific peptides. A general physicochemical evaluation was also conducted, considering possible differences between batches. The antioxidant activity focused mainly on the nitrogen fractions with the shortest-chain peptides. Other parameters were evaluated, including color, weight, size, moisture, dry matter, and texture analysis, which included the whole cheese hardness and the texture profile analysis. The ethanol soluble (EtOH-SN) and non-protein nitrogen (NPN) fractions were selected to evaluate antioxidant activity by the 2,2-diphenyl-1-picrylhydrazyl (DPPH) and 2,2’-azino-bis(3-ethylbenzothiazoline-6-sulfonic acid) (ABTS) methods, total phenol content (TPC), and peptide profiles. Our findings revealed significant differences between batches for NPN ABTS activity and EtOH-SN TPC. Significant differences were observed for water activity, moisture, dry matter, moisture on fat-free basis (MFFB), and pH in the central surface. DPPH and TPC showed a similar behavior, and NPN showed higher values than the EtOH-SN fraction. However, the opposite was observed for ABTS. Significant correlations were found for the biological activities with individual peaks of their corresponding HPLC peptide profiles. Finally, the principal component analysis separated the cheeses according to the batch, mainly due to specific peptides.

## 1. Introduction

Blaťácké zlato cheese has been produced since 1939 in the former Czechoslovakia, in the South Bohemian region that now is part of the Czech Republic. It is similar to the Italian Bel Paese cheese. This cheese is made from pasteurized cow’s milk, and due to its not-pressed nature, whey drainage occurs through gravity, with regular rotation. After this process, the cheese pieces are submerged in brine before the ripening phase begins.

For this kind of cheese, the ripening process is performed at low temperatures, which limits microbial growth [1]. During the ripening period, that could be of several months, rennet enzymes act on the cheese mass leading to significant changes in texture and flavor [1]. Simultaneously, the microbiota action during ripening forms a creamy, yellow, fatty rind that has a slight participation in cheese ripening [1]; nowadays, the surface is covered with a pigment (annatto) to give this yellowish superficial color. At some moment during Blaťácké zlato cheese ripening (about 20 days), cheeses are packed in a heat-shrink plastic film that acts as a ripening bag to continue ripening, additionally, for 4–5 weeks.

Despite its long-standing presence in the Czech market, scientific literature on Blaťácké zlato cheese remains scant. Current databases offer little to no relevant information, and the existing research is often outdated. The nutritional information of the final product has been reported before: 51–53.8% of dry matter [2,3], 27.5 g fat/100 g [4], 48% of fat in dry matter (FDM) [2,5], 1.9 g carbohydrates/100 g, 20.9 g protein/100 g, 840 mg Na/100 g and 2.1 g/100 g NaCl [4]. Additionally, a total of 84.8 g/kg of free amino acids in commercial cheese has been reported by Kabelová, et al. [2], where the most abundant were Glutamine, Proline, Valine, and Leucine.

Early research focused on the general evaluation of different parameters in the Blaťácké zlato cheese ripening and quality, such as the effect of different starter cultures [6,7,8], milk fat content [8], pasteurization conditions [8,9], the origin of microbial rennet [8,10], and salt addition on lactose, free fatty acids, free amino acids, and rheology throughout the ripening [8,9].

More recent studies have been focused on investigating the effectiveness of different films and active packaging materials [11,12] in preserving or enhancing the storage conditions and quality of this cheese [13]. Moreover, few studies have focused on the evaluation and identification of the smear flora, nor on evaluating and identifying the microorganisms causing spoilage. Hanušová, et al. [12] isolated some of these microorganisms to evaluate the efficiency of the active packaging materials.

In recent years, research on dairy products has increasingly focused on their functional properties, particularly the presence of peptides showing biological activity, such as antioxidants, antihypertensive, antimicrobial, etc. Encrypted in caseins, these peptides are released during the ripening by proteolysis.

Antioxidant bioactive peptides are low-molecular-weight amino acid chains that could prevent free radicals or scavenge free radical formation. The length of the chains could go from 2 to 20 amino acid units [14]. The antioxidant activity is usually evaluated in cheese soluble fractions, such as the water-soluble extract (WSN) [15,16,17,18,19,20,21,22], different WSN fractions obtained by gel exclusion chromatography [20], or WSN ultrafiltrate (10 kDa and 3 kDa) [16,20,23]. The most commonly used methods to evaluate the radical scavenging rates are DPPH, ABTS, and TPC [15,16,17,18,21,23]. New approaches in peptidomics focused on evaluating a potential biological activity in silico [19,24].

It has been observed that cheeses showing similarities with Blaťácké zlato cheese present important biological activities, mostly evaluated in the peptide fractions. Antioxidant activity has been reported for Gouda cheese in both the water-soluble fraction [22,25,26] as well as for small peptides (less than 3 kDa) [27], in the water-soluble fraction of Edam [23,26], Emmental [26], and cheddar [20], among other cheeses.

To our knowledge, there are currently few recent studies that include evaluation of the composition of Blaťácké zlato cheese [2], and some of its minor components, such as free amino acids [2], but we have not found published works focusing on the antioxidant capacity of its small peptides.

This study aims to provide new insights into some functional properties of this popular Czech cheese, with a special emphasis on the in vitro antioxidant activity of the nitrogen fractions containing the shortest-chain peptides, while also considering its physicochemical properties.

## 2. Materials and Methods

### 2.1. Samples, Chemicals, and Standards

Three batches of commercial Blat’ácke zlato cheeses were directly acquired from the same producer in 3 different weeks in June 2024 to ensure distinct production batches. For each batch, one whole cheese wheel (1.5 kg), with the intact ripening plastic film package, was obtained to prevent alterations related to cheese cutting and repackaging. The cheeses were transported to the Czech University of Life Sciences Prague, under refrigerated conditions and kept under refrigeration for one day before starting their evaluations. The cheeses were identified with the codes B48B1 (Batch 1), B48B2 (Batch 2), and B48B3 (Batch 3).

Mueller-Hinton Agar, MacConkey Agar, Listeria Selective Agar, and Potato Dextrose Agar were purchased from Oxoid (Basingstoke, UK). Diethyl ether, 2,2-Diphenyl-1-picrylhydrazyl (DPPH), and 6-hydroxy-2,5,7,8-tetramethylchroman-2-carboxylic acid (Trolox) were from Thermo-Scientific (Steinheim, Germany). Gallic acid (GA), potassium persulfate, trifluoroacetic acid, α-cyano-4-hydroxy-cinnamic acid, and 2,2’-azino-bis-(3-ethylbenzothiazoline-6-sulphonic acid (ABTS) were from Sigma-Aldrich Chemie (Steinheim, Germany). Analytical grade methanol, Folin-Ciocalteau reagent, formic acid, HPLC grade acetonitrile, and sodium carbonate anhydrous were from VWR chemicals (Rosny-sous-Bois-CEDEX, France).

### 2.2. Material Conditioning

Prior to the physicochemical evaluation, the cheeses were taken out of the refrigerator and placed at room temperature for two hours. Subsequently, all the pieces were weighed to evaluate their uniformity, followed by a comprehensive evaluation encompassing different physicochemical, compositional, and functional parameters.

#### 2.2.1. Physicochemical Properties

Hardness was evaluated in the whole cheese. Next, the cheeses were cut into two parts horizontally at half-height to expose the cheese’s central surface. pH was evaluated by the average of five different measurement points of the cheese surface and center using an Eco pHTestr2 (Thermo-Scientific, Oxford, UK).

##### Color

The color parameters were determined only in the cheese central surface as the mean of 5 measure points in different points of the center surface by using a CM-23d spectrophotometer (Konica Minolta Holdings, Inc., Tokyo, Japan) with an illuminant D65, 10° viewing angle and an 8 mm orifice, and a software SpectraMagic™ NX (version CM-S100W). CIELAB color space coordinates L* (lightness), a* (green-red axis), and b* (blue-yellow axis) were recorded for each cheese; additionally, the polar coordinates Chroma (C*) and hue° (hue angle) were calculated (Equations (1) and (2)).

(1) $$C^{*}=\sqrt{{a^{*}}^{2}+{b^{*}}^{2}}$$

(2) $$h{}^{\circ}=\mathrm{atan}\left(\frac{b^{*}}{a^{*}}\right)$$

Next, cheeses were portioned, and about 500 g of each cheese, including rind and core, was cut into smaller fractions and mixed to get a uniform sample. Portions for moisture, water activity (aw), and fat determinations were separated, and the rest were portioned (about 20 g per portion), placed in aluminum foil and tightly closed bags, and frozen (−18 °C) until analysis.

##### Moisture and Fat Content, and Water Activity

Moisture (AOAC 934.01) and fat (AOAC 920.85) and fat contents were determined using the AOAC methods [28], while aw was measured with an AW-Therm M (Rotronic AG, Bassersdorf, Switzerland) meter with a precision of ±0.005. These analyses were made in duplicate.

The moisture on a fat-free basis MFFB (%) and the fat in dry matter FDM (%) were calculated to classify the cheeses using Equations (3) and (4) [29,30].

(3) $$MFFB\%=\frac{Weight\;of\;moisture\;in\;cheese}{Weight\;of\;cheese-Weight\;of\;fat\;in\;cheese}*100$$

(4) $$FDM\%=\frac{Weight\;of\;fat\;in\;cheese}{Weight\;of\;cheese-Weight\;of\;water\;in\;cheese}*100$$

#### 2.2.2. Textual Parameters

The textural parameters were evaluated first in the whole cheese by penetration (hardness) and next on pieces of the cheese by the texture profile analysis (TPA). In both cases, a texturometer (Shimadzu EZ-SX, Shimadzu, Kyoto, Japan) was used and evaluated using Trapezium software ver. 1.5.6 (Shimadzu).

The hardness (maximum force developed during the penetration, in Newtons, N) of the original cheese blocks was assessed by inserting a 15 mm diameter needle probe to a depth of 20 mm at a speed of 5 mm/s. The register of the force started when the probe had the first contact with the sample surface (overcoming a force of 0.15 N).

For the TPA, a 20 mm cylindrical probe was used. Cheese pieces were cut to have the same dimensions (10 mm × 10 mm × 4 mm). The compression ratio used was 70% deformation from the initial height at a rate of 30 mm/s.

### 2.3. Isolation and Characterization of Microorganisms

#### 2.3.1. Cultivation of Microorganisms

Ten grams from the surface (depth = 5 mm) and ten grams from the center (depth = 3 cm) of the tested cheese were each aseptically homogenized in 90 mL of sterile saline solution and ten-fold serially diluted (continuous transfer of 1 in 9 mL). For inoculation, 10 µL of the appropriately diluted samples were plated using the spread plate method with a sterile inoculation loop (Avantor, Stribrna Skalice, Czech Republic) on the following media: Mueller-Hinton Agar (non-selective), MacConkey Agar (Enterobacteriaceae and gram-negative bacteria), Listeria Selective Agar (*Listeria monocytogenes*), and Potato Dextrose Agar (fungi, yeasts, and molds). The plates were subsequently cultivated under aerobic conditions at 25 °C (Potato Dextrose Agar) and 37 °C (other media) for the respective 5 and 1–2 days. After the growth, each morphologically distinct colony was counted by the manual colony counter CC-200 (Cole-Parmer Instrument Company, Cambridgeshire, UK).

#### 2.3.2. Culture Isolation and Identification

Standard procedure recommended by Bruker Daltonics (Billerica, MA, USA) for the MALDI-TOF mass spectrometry identification was used in this experiment. A small amount of biological material of fresh microbial culture from each morphologically distinct colony was taken from the growth medium and directly spread on individual spots of the steel MALDI target plate. Each sample spot was overlaid with 2 µL of matrix solution (saturated 1% solution of α-cyano-4-hydroxy-cinnamic acid in 50% acetonitrile, 47.5% water, and 2.5% trifluoroacetic acid) and air-dried for 15 min before analysis. For the potentially isolated fungi, the sample was consecutively extracted in water, 96% ethanol, and a 1:1 (*v*/*v*) solution of 70% formic acid and 100% acetonitrile, centrifuged, and 1 µL of supernatant was overlaid before adding the matrix solution. To identify the microorganisms, the raw spectra obtained for each isolate were imported into the BioTyper software, version 2.0 (Bruker Daltonik, GmbH, Bremen, Germany) and analyzed without any user intervention.

### 2.4. Peptides Fractionation

For the evaluation of the antioxidant activity, EtOH-SN and trichloroacetic acid soluble or non-protein nitrogen (NPN) crude nitrogen fractions were selected since they contain only shorter chain peptides. Both of the selected fractions contain small peptides (2–20 residues) and free amino acids; nevertheless, in the recovery of the EtOH-SN fraction, more of the low molecular-weight peptides are precipitated, giving slight differences between the two fractions.

To obtain these nitrogen fractions, cheeses were fractioned using the methods described by Christensen et al. [31] and Kuchroo and Fox [32]. Briefly, 10 g of cheese was dispersed in 20 mL of distilled water using an ultraturrax (IKA 18, IKA Labor Technik, Staufen, Germany) at 20,000 rpm for 5 min, next incubated at 40 °C for 1 h, followed by 5 min dispersion with the ultraturrax and centrifugation for 30 min at 4 °C and 3000× *g*. The fat upper layer was discarded, obtaining defatted cheese suspensions (DS) that were resuspended by manual agitation. The samples were kept at −18 °C until fractionation.

Subsequently, 3 mL of DS was added with 6 mL of deionized water, and pH 4.6 was adjusted with 1N HCl, followed by centrifugation for 30 min at 4 °C and 3000× *g*. The soluble fraction corresponded to the acid-soluble nitrogen fraction (ASN). For the EtOH-SN, 3 mL of ASN was added with absolute ethanol to achieve a final concentration of 70% ethanol (*v*/*v*) and left overnight at room temperature and subsequently centrifuged for 30 min at 4 °C and 3000× *g*, and the supernatant recovered (EtOH-SN) was kept at −18 °C until analysis.

Finally, for the NPN fraction, 5 mL of DS was added with 3 mL of deionized water and 2 mL of trichloroacetic acid (TCA) at 60% (*v*/*v*) to a final concentration of 12%. TCA. After 30 min centrifugation at 4 °C and 3000× *g*, the NPN fraction was separated and kept at −18 °C until analysis.

### 2.5. Nitrogenous Fractions Analysis by RP-HPLC

The peptide profiles of the EtOH-SN and NPN were obtained by direct analysis of the fractions using an RP-HPLC. Before the analysis, the samples were filtered through a 0.45-μm filter (PTFE Syringe Filter, J.T Baker, VWR International, Radnor, PA, USA). The reverse-phase high-performance liquid chromatography (RP-HPLC) was carried out in an Agilent Technologies 1200 series (Palo Alto, CA, USA) coupled with a DAD. Compound separations were done at 30 °C using an InfinityLab Poroshell 120 EC-C18 (3.0 × 150 mm, 2.7 µm, Agilent Technologies, Palo Alto, USA). A gradient solvent flow of 0.500 mL/min was established using solvent A: Water HPLC-grade with 0.1% of TFA and solvent B: Methanol HPLC-grade with 0.1% of TFA. The elution program was as follows: initially 100% of A for 5 min, next a linear gradient to pass from 100% A to 51% A at 49 min, afterward, from 51% A to 20% A at 54 min, and to 0% A at 57 min, and maintained until 60 min. The sample volume was 10 μL. The wavelength in the DAD was 215 nm with a resolution of 2 nm. Data acquisition was done using the OpenLab software (Agilent Technologies). Results were reported as relative area percentage. To classify the peptides as hydrophilic or hydrophobic, we consider the gradient reported by Gonzalez De Llano et al. [33] and compare it with our gradient instead of the chromatogram retention time. The hydrophilic peaks corresponded to a range from 0 min to 20 min (from 0% to 17% of methanol), while the hydrophobic peptides corresponded to a range from 20 min to 60 min (from 17% methanol to 100% methanol). The ratio between the two groups of peptides was also calculated.

### 2.6. In Vitro Bioactivity

#### 2.6.1. Antioxidant Capacity

Bioactivity was evaluated in the EtOH-SN and NPN fractions. All the determinations were done using a UV-1600PC spectrophotometer (VWR, Radnor, PA, USA).

DPPH was measured using the method described by Brand-Williams et al. [34] with slight modifications. 100 µL of sample/standard was added to 900 µL of a 150 µM DPPH solution in 80% methanol. Absorption at 520 nm was recorded after a 30-min reaction.

ABTS was measured using the method described by Re et al. [35] with slight modifications. A stock ABTS solution containing 7 mM ABTS and 2.4 mM potassium persulfate solution was prepared. After 16 h of agitation in the dark, a working solution was prepared by diluting 1:100 (*v*/*v*) of the stock ABTS solution in methanol. The final working solution was adjusted to a 0.700 ± 0.01 absorbance at 734 nm. For samples or standards analysis, 100 µL of sample/standard was added to 900 µL of ABTS working solution. Absorbance at 734 nm was recorded after 30 min of reaction.

Trolox was used as an external standard for both methods, and a calibration curve was constructed (r^2^ = 0.9934 and r^2^ = 0.9846, respectively). Results were expressed as micromoles of Trolox equivalents per gram of fresh sample (µM TE/g f.w.).

#### 2.6.2. Total Phenolic Content (TPC)

TPC was measured using the Folin–Ciocalteau method described by Singleton and Rossi [36] with slight modifications. For samples or standard analysis, 0.25 mL of the sample/standard was added to 1 mL of distilled water and 0.25 mL of the diluted (1:1 *v*/*v* with water) Folin–Ciocalteau reactive, mixed, and let to stand for 6 min. Next, 2 mL of distilled water and 2.5 mL of a 7% sodium carbonate solution were added and left to react for 90 min at room temperature in the dark. Finally, absorbance was measured at 760 nm. A Gallic acid calibration curve was constructed (r^2^ = 0.9984). Results were expressed as milligrams of gallic acid equivalents (GAE) per gram of fresh sample (mg GAE/g f.w.)

### 2.7. Statistical Analysis

All the statistical analyses were carried out using the STATISTICA software 14.1 (TIBCO Software Inc., Palo Alto, CA, USA). First, basic descriptive statistical analysis was carried out for all the physicochemical and compositional parameters. Next, an analysis of variance (ANOVA) was done to evaluate the uniformity of the batches and, if that was the case, identify the parameters showing significant differences among them. Additionally, a simpler linear correlation analysis (Pearson’s *r*) was carried out to identify significant correlations (*p* < 0.05) between cheese fraction peptide profiles (individual peaks in the chromatograms) and the corresponding fraction biological activity.

## 3. Results and Discussion

### 3.1. Physicochemical Parameters

**Figure 1:**
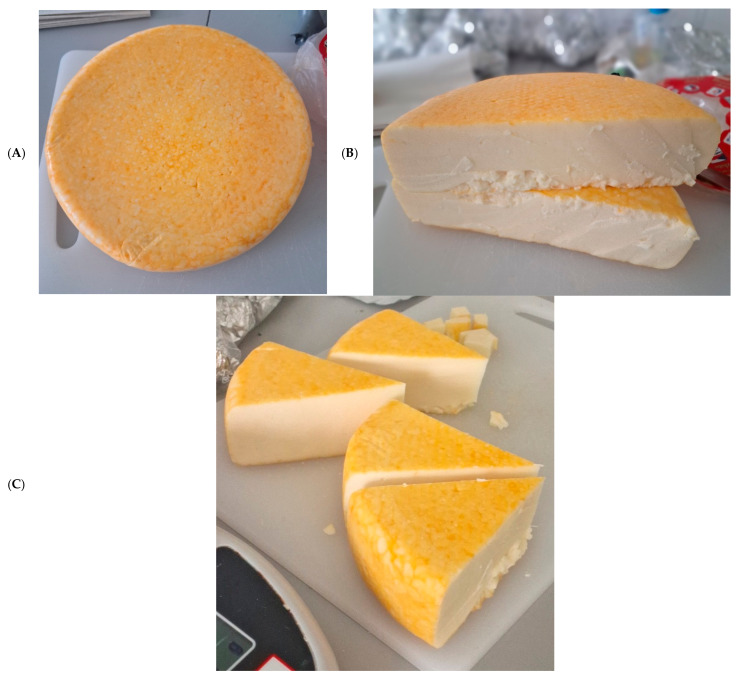
Blat’ácke zlato cheeses: (**A**) External appearance; (**B**) Horizontal cut showing the internal appearance, and (**C**) Portions cut showing paste homogeneity.

Visually, the Blat’ácke zlato cheese samples analyzed presented a homogeneous yellow color on the surface, as in the center, without visible defects (Figure 1).

Blat’ácke zlato cheeses exhibited uniformity in both weight and color, showing no significant differences among different batches (*p* > 0.05) (Table 1). The average weight of the cheeses was 1457 g with a consistent diameter of 19 cm, and they displayed a yellow-white color in the cheese center (measured by L*, a*, and b* parameters) with no significant variations (Table 1).

Significant differences (*p* < 0.05) among batches were observed for aw, moisture, dry matter, and MFFB, while the other general parameters did not show significant differences (Table 1).

The dry matter content was higher than previously reported values, which ranged from 51–53.8% [2,3,5,37]. In contrast, the fat content did not show significant differences among batches, and fat in wet basis values were similar to those reported before for this kind of cheese [3,4,5,37], as well as fat in dry bases [2,4]. However, both the dry and wet basis fat content were lower than those reported for similar cheeses such as Bel Paese cheese and Butterkäse cheeses [38]. The values obtained for MFFB and FDM (Table 1) made it possible to classify the analyzed cheeses as categorized as semi-hard/soft and full fat (61.6% and 48%, respectively) according to the criteria outlined in the CXS 283-1978 [30]. FDM values were according to previously reported values for a similar type of cheese [4].

Texture evaluation showed significant differences in the whole cheese hardness and the TPA parameters, except for adhesiveness (Table 1). The differences in hardness may be attributed to the non-ideal uniformity of the cheese block structure, which is common for any batch of real food materials [39].

### 3.2. Microbiological Analysis

The results of the microorganisms’ identification by MALDI-TOF MS made it possible to identify four strains of *Lactococcus lactis* with high certainty (Log(score) 2.0–2.3): *Lactococcus lactis* DSM 4366 DSM, *Lactococcus lactis* IBS_MS_6 IBS, *Lactococcus lactis* ssp. *lactis* 3C1_QSA IBS, and *Lactococcus lactis* ssp. *lactis* DSM 20481T DSM. Two different strains were identified on the cheese surface, and the two remaining were in the cheese center. *L. lactis* is a microbial species extensively used in producing dairy products as a starter bacterium, including Saint-Paulin cheese [40]. One strain of *Staphylococcus equorum* was identified on the cheese surface, with medium certainty only (Log(score) 1.7–2.0). *S. equorum* is a commensal species frequently detected in fermented foods such as cheese surfaces [41]. No grown microbial colonies were observed on selective media (MacConkey Agar, Listeria Selective Agar, and Potato Dextrose Agar) after the incubation time. The absence of yeasts, molds, enterobacteria, and listeria reflects normal hygienic conditions and proper handling. Since Blaťácké zlato is not designed as a mold-ripened cheese, fungi usually represent post-processing contamination, particularly in those with compromised packaging and prolonged storage [12]. Therefore, we assume that the number of spores potentially present on the surface did not permit viable transfer to agar media for cultivation.

### 3.3. Peptide Profiles

A total of 122 peaks were identified in the EtOH-SN and NPN fractions (Table 2), and they were coded as Px according to their retention time: 64 peaks in the EtOH-SN fraction and 91 in the NPN fraction. Of the identified peaks, 34 peaks were present in both fractions. Blat’ácke zlato cheeses presented uniformity between batches since none of the peaks presented significant differences in the integrated areas. Even though both fractions correspond to the small chain peptides, their profiles were different. The NPN fraction presented a high proportion of hydrophilic peptides (HI), while for the EtOH-SN, the hydrophobic peptides presented the highest proportion (HO).

### 3.4. Antioxidant Activity

NPN and EtOH-SN fractions were selected to determine their in vitro biological activity since they contain short-chain peptides (3–20 units). Nevertheless, these fractions present differences in the profile of the precipitated peptides. We confirmed these differences in the peptide profiles of those fractions (Table 3).

Analysis of variance (ANOVA) showed significant differences in the bioactivity of the fractions (Table 3). Antioxidant DPPH activity and TPC were significantly superior in the NPN (*p* = 0.000) fraction, while ABTS was in the EtOH-SN (*p* = 0.000) fraction. On the other hand, significant differences between batches were observed for ABTS and TPC depending on the cheese fraction. In contrast, for DPPH, none of the fractions showed significant differences among batches (Table 1). ABTS showed significant differences between batches in the NPN (*p* = 0.003), while TPC presented significant differences in the EtOH-SN fraction (*p* = 0.003).

Antioxidant DPPH activity values in NPN were higher than those reported for other semi-hard/soft cow’s milk cheeses [42,43]. In our study, the percentage of DPPH discoloration was 55.76 ± 2.98%, while Pritchard et al. [42] reported a maximum of 15% discoloration in the cheddar cheese water-soluble extract (WSE), and Timón et al. [43] reported a maximum of 7.63% discoloration in WSE of cow’s milk cheeses made with different coagulants. ABTS in NPN was superior to that reported for the water-soluble fraction (WSF) of Caciocavallo cheeses [44].

Phenolic compounds in cheeses are primarily associated with the animal diet since they are transferred to milk [45] or to some ingredients added during cheese production. Even though annatto is used in the analyzed cheeses, we only partially attribute the differences in antioxidant activity and the TPC to this ingredient since these compounds are hydrosoluble and must be present in both fractions in really similar concentrations. Besle et al. [45] reported different polyphenols in cow’s milk; the specific compound and its concentration depended strongly on the forage used for cow feeding. Nevertheless, TPC has been reported in cheeses without added ingredients. Our results were inferior to the TPC reported for Vastedda valle del Belìce PDO, a semi-hard Italian cheese [46].

### 3.5. Correlation Analysis

The correlation analysis revealed significant correlations (*p* < 0.05) between the antioxidant activity of the nitrogen fractions and the corresponding individual peptides on the profiles obtained by HPLC (Table 3). Table 4 lists the individual peptides that display at least one significant correlation with the biological activities.

NPN showed significant negative correlations between DPPH antioxidant activity with peaks P38, P58, P77, and P111, with the ABTS antioxidant activity with peaks P2, P18, P21, and P25, and for TPC with P5, P7, P34, P37, P71, P98, and P102. On the other hand, the EtOH-SN showed significant correlations between DPPH antioxidant activity with peaks P86 and P91, a significant correlation between ABTS antioxidant activity only with P108, and with the TPC with peaks P70 and P79.

## 4. Conclusions

Based on the obtained physicochemical results, Blaťácké zlato cheese would fall under the classification of semi-hard/soft and full-fat cheese. The characterization showed that even though there were no statistical differences between batches for color, weight, and size, there were statistical differences between texture and MFFB. The nitrogen fractions presented in vitro antioxidant activity, determined as DPPH and ABTS, and the presence of phenolic content (measured as TPC), and these biological activities could be correlated with specific peaks in the peptide profiles. PCA made it possible to classify the cheeses according to the production batch, principally by two peaks or peptides present in nitrogen fractions. The characterization of this product can help better understand the functional properties of this popular cheese. Further studies are needed for a deeper characterization, including the evaluation of other biological activities, the effect of seasonality, and the effect of ripening time, among others.

## Figures and Tables

**Table 1:** Physicochemical characterization of Blaťácké zlato cheese.

Parameter	*p*-Value ^a^	Valid N	Average ± SD
**General parameters**			
Weight (g)	0.998	3	1457.5 ± 101.01
Diameter	---	3	19.0 ± 0.0
aw	0.000	6	0.938 ± 0.0052
Moisture (%)	0.034 *	6	45.29 ± 1.519
Dry matter (%)	0.000 ***	6	54.71 ± 1.519
Fat Dry Basis (%)	0.129	6	48.07 ± 5.890
Fat Wet Basis (%)	0.187	6	26.28 ± 3.181
MFFB (%)	0.040 *	6	61.56 ± 3.434
FDM (%)	0.129	6	48.07 ± 5.890
pH Surface	0.5861	15	5.347 ± 0.146
pH Center	0.019 *	15	5.200 ± 0.193
**Texture** ^α^			
Hardness (N)	0.008 **	9	4.861 ± 0.887
**TPA ^β^**			
Hardness (N)	0.000 ***	30	20.78 ± 9.653
Cohesiveness	0.000 ***	30	0.2254 ± 0.2630
Adhesiveness (J)	0.058	30	−0.0014 ± 0.00285
Adhesiveness force (N)	0.000 ***	30	−0.5874 ± 0.3793
**Color**			
*L* *	0.198	16	85.84 ± 3.073
*a* *	0.759	16	0.939 ± 0.310
*b* *	0.380	16	17.18 ± 1.768
Chroma	0.377	16	17.21 ± 1.780
hue	0.537	16	86.89 ± 0.940
**DPPH ^b^**			
NPN ^d^	0.644	6	657.90 ± 39.15
EtOH-SN ^e^	0.307	6	278.33 ± 91.33
**ABTS ^b^**			
NPN ^d^	0.003 **	6	61.05 ± 29.47
EtOH-SN ^e^	0.139	5	1587.28 ± 356.19
**TPC ^c^**			
NPN ^d^	0.285	6	153.16 ± 33.70
EtOH-SN ^e^	0.003 **	6	61.05 ± 29.47

^a^ Significance at *p* ≤ 0.05 is indicated with *, at *p* ≤ 0.01 with **, and at *p* ≤ 0.001 with ***. ^b^ Expressed as TE μM/g cheese. ^c^ Expressed as μg GAE/g cheese. ^d^ NPN: Nonprotein nitrogen. ^e^ EtOH-SN: Ethanol-soluble nitrogen. ^α^ Evaluation as penetration force. ^β^ Evaluated by texture profile analysis.

**Table 2:** Peptide profile of Blaťácké zlato cheese nitrogen fractions.

RT [min]	Peak Code	EtOH-SN ^a^	NPN ^b^	RT [min]	Peak Code	EtOH-SN ^a^	NPN ^b^
		Mean ± SD	Mean ± SD			Mean ± SD	Mean ± SD
0.711	P2	0.19 ± 0.06	0.15 ± 0.07	42.788	P70	0.06 ± 0.05	
0.981	P4	0.40 ± 0.09	0.10 ± 0.06	43.971	P72	0.10 ± 0.04	0.01 ± 0.01
1.181	P5	2.18 ± 0.39	0.24 ± 0.08	44.212	P73	0.08 ± 0.04	
1.349	P7	2.67 ± 0.36	1.05 ± 0.58	44.902	P75		0.03 ± 0.02
1.876	P9	6.76 ± 1.68	2.31 ± 0.53	45.406	P76	0.07 ± 0.03	0.002 ± 0.001
2.384	P10	0.07 ± 0.07	0.12 ± 0.03	46.249	P77		0.20 ± 0.06
2.663	P13	0.21 ± 0.21	72.93 ± 8.39	46.663	P78	0.31 ± 0.24	0.10 ± 0.01
3.990	P15		7.91 ± 13.57	47.299	P81	0.33 ± 0.08	0.07 ± 0.01
9.456	P16	0.91 ± 0.49	0.13 ± 0.14	48.204	P82		0.04 ± 0.03
11.248	P17	0.14 ± 0.08	0.00 ± 0.00	48.907	P83		0.99 ± 0.41
11.160	P18		0.05 ± 0.01	49.361	P84	2.24 ± 2.51	
11.636	P19	0.75 ± 0.31	0.17 ± 0.04	49.714	P85	1.30 ± 0.28	
13.289	P20	1.53 ± 1.65		50.136	P86	1.40 ± 0.52	0.86 ± 0.29
15.104	P21		0.005 ± 0.003	50.547	P87	1.92 ± 2.47	
18.149	P24	1.27 ± 0.16		51.199	P88		0.12 ± 0.06
19.815	P26		0.042 ± 0.002	53.579	P93	2.17 ± 0.83	
21.308	P30	0.31 ± 0.26		53.337	P94		0.35 ± 0.22
22.642	P32	0.05 ± 0.03	0.19 ± 0.10	53.614	P95	0.25 ± 0.09	
24.334	P34	14.08 ± 2.85	2.21 ± 0.36	53.894	P97		1.35 ± 0.47
26.247	P36		0.02 ± 0.02	54.244	P98	8.06 ± 1.28	0.12 ± 0.04
27.404	P39		0.02 ± 0.00	54.521	P99	0.18 ± 0.01	0.01 ± 0.00
28.593	P42	0.59 ± 0.19	0.04 ± 0.00	55.137	P102	0.17 ± 0.14	0.02 ± 0.00
33.834	P52	0.17 ± 0.08		55.258	P104	0.19 ± 0.04	
34.209	P53		0.01 ± 0.00	55.696	P107	5.14 ± 2.83	0.01 ± 0.01
35.879	P56		1.50 ± 0.57	56.176	P108	0.80 ± 0.47	0.02 ± 0.01
36.399	P57	8.62 ± 1.11		56.583	P109	0.14 ± 0.03	0.01 ± 0.01
37.190	P58		0.32 ± 0.09	56.862	P110	7.94 ± 2.67	0.12 ± 0.06
37.839	P60	0.12 ± 0.02	0.02 ± 0.01	57.326	P113	2.55 ± 2.97	
38.176	P61	2.59 ± 0.45	0.01 ± 0.01	57.481	P114	0.10 ± 0.05	
38.564	P62		0.02 ± 0.03	57.662	P115		1.34 ± 0.95
39.812	P63		0.002 ± 0.001	57.736	P116	4.05 ± 1.13	1.66 ± 0.56
40.583	P64	0.12 ± 0.18		58.061	P117	0.36 ± 0.22	
41.747	P66		0.01 ± 0.01	59.166	P119	0.21 ± 0.07	0.04 ± 0.03
42.087	P67		0.05 ± 0.01	59.811	P120	0.32 ± 0.12	0.15 ± 0.14
42.587	P69	0.32 ± 0.39		59.996	P121	0.28 ± 0.18	
				EtOH-SN ^a^		NPN ^b^	
Total Hydrophilic peptides (HI)				18.63 ± 0.58		86.12 ± 3.85	
Total Hydrophobic peptides (HO)				72.13 ± 6.04		13.86 ± 3.85	
Ratio HO/HI				3.87 ± 0.22		0.16 ± 0.05	

^a^ EtOH-SN: Ethanol soluble nitrogen ^b^ NPN: Nonprotein nitrogen.

**Table 3:** Analysis of Variance (ANOVA) of the biological activity of nitrogen fractions from Blaťácké zlato cheese.

Parameter	*p*-Value ^a^	Fraction	
		NPN ^µ^	EtOH-SN ^π^
DPPH ^α^	0.000 ***	657.90 ^b^	287.64 ^a^
ABTS ^α^	0.000 ***	61.05 ^a^	1587.28 ^b^
TPC ^β^	0.000 ***	153.16 ^b^	59.21 ^a^

Significance at *p* ≤ 0.001 is indicated with ***, Means with different letters within the same row are significantly different (*p* < 0.05). ^α^ Expressed as TE μM/g cheese. ^β^ Expressed as μg GAE/g cheese. ^µ^ NPN: Nonprotein nitrogen. ^π^ EtOH-SN: Ethanol-soluble nitrogen.

**Table 4:** Correlation analysis of the peptides with the biological activity of nitrogen fractions from Blaťácké zlato cheese.

Peak Code	DPPH		ABTS		TPC	
	NPN	EtOH-SN	NPN	EtOH-SN	NPN	EtOH-SN
P2	−0.009		−1.000 **		−0.679	
P5	−0.786		−0.632		−0.998 *	
P7	−0.757		−0.667		−1.000 **	
P18	0.066		0.999 *		0.720	
P21	0.030		1.000 **		0.694	
P25	−0.046		−1.000 **		−0.705	
P34	−0.783		−0.636		−0.998 *	
P37	−0.785		−0.634		−0.998 *	
P38	0.998 *		−0.044		0.698	
P58	−1.000 **		−0.009		−0.734	
P70		−0.694		0.973		1.000 **
P71	0.744		0.681		1.000 **	
P77	−0.999 *		−0.067		−0.773	
P79		0.725		−0.962		−0.998 *
P86		−0.999 *		0.539		0.705
P91		−0.999 *		0.468		0.644
P98	−0.714		−0.713		−0.999 *	
P102	−0.749		−0.676		−1.000 **	
P108		−0.565		0.998 *		0.989
P111	−0.998 *		0.040		−0.701	

Bold values present a significance, at *p* ≤ 0.05 are designed with *, and at *p* ≤ 0.01 with **.

## Data Availability

Data will be made available on request.
